# In silico prediction of GRP78-CRIPTO binding sites to improve therapeutic targeting in glioblastoma

**DOI:** 10.1038/s41598-025-00125-z

**Published:** 2025-05-13

**Authors:** Mahmoud E. Rashwan, Mahrous R. Ahmed, Abdo A. Elfiky

**Affiliations:** 1 Physics Department, Faculty of Science, Sohag University, Sohag, 82524 Egypt; 2 Biophysics Department, Faculty of Science, Cairo University, Giza, 12613 Egypt

**Keywords:** GRP78, Cripto, Glioblastoma multiforme, Computational biophysics, Protein–protein docking, molecular dynamics simulation, Biophysics, Computational biology and bioinformatics

## Abstract

Glioblastoma multiforme (GBM) is one of the most malignant tumors in central nervous system (CNS) tumors. The glucose-regulated protein 78 (GRP78) and CRIPTO (Cripto-1), a protein that belongs to the EGF-CFC (epidermal growth factor cripto-1 FRL-1 cryptic) family, are overexpressed in GBM. A complex between GRP78 SBDβ (substrate binding domain beta) and CRIPTO CFC domain was reported in previous studies. This complex activates MAPK/AKT signaling, Src/PI3K/AKT, and Smad2/3 pathways which is a reason for tumor proliferation. In this work, we study how the two proteins form the complex figuring out binding sites between GRP78 and CRIPTO utilizing computational biophysics and bioinformatics tools, such as protein–protein docking, molecular dynamics simulation and MMGBSA calculations. Haddock web server results of 4 regions from the CFC domain (region1 (− 70.4), region2 (− 78.7), region3 (− 74.2), region4 (− 86.8)) with selected residues of the SBDβ are then simulated for 100 ns MDS then MMGBSA were calculated for the four complexes. The results reveal the stability of the complexes with binding free energy (complex1 (− 15.07 kcal/mol), complex2 (− 59.78 kcal/mol), complex3 (− 81.92 kcal/mol), complex4 (− 126.26 kcal/mol). All these findings ensure that GRP78 SBDβ associates with the CRIPTO CFC domain, and the binding sites suggested make stable interactions between the proteins.

## Introduction

Glioblastoma multiforme (GBM) is classified as a WHO grade IV brain tumor and is the most common and the most aggressive of the primary malignant tumors of the human brain. According to some estimates, it accounts for 14.5% of central nervous system tumors and about 50% of the malignant cases within this group^1^. The GBM is known to increase with age, being most rampant among the aged 65–84 years, and it is also more prevalent among men and Caucasians compared to all other population groups^2,3^. According to some of the reports that estimated the incidence of GBM, there is inconsistency as the figures provided range between approximately 3.19 incidents per 100,000 persons per year to 3.3^4,5^. In terms of how they are usually presented, GBM presents with vague symptoms, such as persistent headaches, cognitive changes, and alterations in personality, which may all pose a challenge to timely diagnosis and management^6^. These multi-source differences should be understood in order to achieve the common goals of preventing and treating the disease in different populations. This formidable tumor arises primarily from astrocytic glial cells, which play crucial roles in supporting neural function. Another research states that GBM may begin from various cell sources including neural stem cells (NSCs) or oligodendrocyte precursor cells (OPC), resulting in different tumor characteristics and behaviors that are detected in experimental models^7,8^. There are two main types of GBM: primary and secondary. The primary form arises spontaneously and accounts for the majority of cases, while secondary GBM begins in pre-existing lower-grade astrocytoma^9,10^. With recent discoveries in molecular biology, it has now become possible to subclassify GBM into four molecular subtypes: classical, mesenchymal, proneural, and neural, which provide a better understanding of the tumor biology and cellular processes and potentially provide strategies for targeted (personalized) therapy^6^. Understanding the progression of the cases is critical when it comes to management and looking for methods of treatment. The median overall survival has been seen to be around 15 months which is very concerning, on average^10^. Ionizing radiation is suggested to be an additional risk for GBM development as certain studies have shown that individuals with such exposure have an increased probability of developing brain neoplasms^11^. It is necessary to understand the complexity of GBM with respect to its cell of origin, clinical presentation, and response to treatment, for the development of newer vectors that could alleviate the problems posed by this aggressive and often fatal neoplasm.

Glucose-regulated protein 78 (GRP78), also known as HSPA5 or immunoglobulin heavy chain binding protein (BiP), is a pivotal member of the Heat Shock Protein 70 (HSP70) family. GRP78, in all eukaryotic cells, is most commonly found on the Endoplasmic Reticulum (ER)^12^. This multifunctional protein is involved in the folding and proper assembly of proteins. It also prevents the exodus of incorrectly folded proteins or protein subunits, thus maintaining cellular homeostasis and functionality^13^. The Unfolded Protein Response (UPR) kicks in when cells face stress from unfolded or misfolded proteins building up in the ER lumen. Cancer researchers have zeroed in on this response because it helps tumors survive and resist treatments in cancers like astrocytoma and melanoma. GRP78 has caught scientists’ eyes as it plays a central role in controlling the UPR^14,15^. Studies have linked high levels of GRP78 to more aggressive glioblastoma (GBM) tumor cells, making it a promising target for new therapies^16^.

A new study sheds light on how Cripto-1 (also named CRIPTO) plays a key part in boosting stemness, growth, invasion, migration, and angiogenesis (blood vessel formation) in human glioblastoma (GBM) cells in the U87 cell line^17^. Higher levels of Cripto-1 also have a link to many cancers such as those in the breast, colon, lung, and brain^18–21^. Cripto-1 (CRIPTO) belongs to the EGF-CFC (epidermal growth factor cripto-1 FRL-1 cryptic) family and has a crucial role in keeping embryonic stem cells alive in many species, humans included^18,22–24^. It acts as a co-receptor for key TGF-β family ligands such as (Nodal, GDF1, and GDF-3) while forming a complex with ALK4 (Activin receptor types I and II)^19,25,26^. Cripto-1 influences many pathways. It boosts cell growth and controls movement in tumor-related endothelial cells. This happens when it interacts with substances like Glypican-1 and the heat shock protein GRP78^27^. These interactions can mess up cell sticking and turn on pathways such as MAPK/AKT and SMAD2/3 phosphorylation^28,29^. This suggests that pathways active during growth might start up again as tumors get worse^18^. Because of this, Cripto-1 shows promise as a sign of tumor growth. It’s important in both early growth and cancer studies^20^.

Cripto-1 (CRIPTO) was found to form a complex with csGRP78 in various types of cancers such as embryonal carcinoma, prostate cancer, and liver cancer^28,30,31^. The CRIPTO/Grp78 complex plays a significant role in modulating various signaling pathways, notably impacting MAPK/AKT signaling and the Src/PI3K/AKT pathways, inducing cell proliferation, plasticity, and resistance to apoptosis. Besides that, this complex also acts in antagonism with Activin/Nodal/TGF-β signaling. These interactions reflect the complexity of cellular signaling networks and point toward the cripto-1/GRP78 complex as a critical target for therapeutic intervention in related diseases^25,32^.

Computational biophysics integrated with bioinformatics has reduced time and cost drastically in drug discovery. In-silico approaches have transformed our capacity to explore disease mechanisms on many scales of biology. Classical workflows integrating predictive algorithms SIFT (Sorting Intolerant From Tolerant) and PolyPhen (Polymorphism Phenotyping), molecular dynamics (MD) simulations and molecular docking have been successful in characterizing protein mutations such as OCA2’s P protein (R305W mutation) and PI3Kα (E542K/E545K mutations), identifying destabilization patterns and modified binding affinities^33,34^. These methods have been used to study PLK1’s W414F mutation, where stringent in-silico screening and MD simulations showcased how structural mobility affects cell cycle regulation^35^.

The field has since expanded from protein-coding mutations to investigate regulatory processes. A review of miR-TS-SNPs in CEP genes highlighted the manner in which mutations in the 3’UTR can disrupt centrosome function through miRNA-mediated translational control^36^. Similarly, the S97C mutation in CK1δ was reported to reduce ATP-binding affinity by conformational changes, correlating structural alterations with breast cancer pathogenesis^37^. These investigations collectively demonstrate the manner in which computational methods can bridge gaps between genotype and phenotype at regulatory levels. Recent advances now make direct drug discovery applications possible, as evidenced by the identification of triazole-derived PDE10A inhibitors of neurological disorders. This study combined molecular docking, MD simulations, and advanced free energy calculations (MM-PBSA, steered MD) to characterize inhibitor binding, exemplifying the translational potential of computational biophysics^38^.

This research paper identifies molecular dynamics simulation and protein–protein docking techniques helpful in the identification of effective ligands^39,40^. Websites, such as Protein Data Bank and PubChem provide the structures of various proteins and ligands^41,42^. The use of bioinformatics tools enables research into protein structures and aids in understanding the molecular basis for many biological processes. In this respect, the latest developments in computational biology have dramatically changed the ability to predict protein structures with great accuracy via AlphaFold2. It finally brings a big step towards the enhancement of docking studies and interaction predictions, coming out with more reliable and comprehensive results^43^. Now, the combination of structural biology with computational analysis may pinpoint critical molecular interactions and conserved regions in proteins, supplying a robust foundation for new drug candidate discovery. Interdisciplinary approaches in drug design thus have taken a front-row seat and allow novel avenues in disease targeting.

## Materials and methods

CFC domain of CRIPTO is a cysteine-rich domain and has previously been implicated in molecular recognition processes. GRP78, a very conserved molecular chaperone, is operationally characterized by its ability to bind a very diverse range of substrates, often in terms of general structural or chemical properties, rather than high sequence conservation. This substrate promiscuity is likely an evolutionary adaptation of GRP78 to bind a range of partners, including misfolded proteins, peptides, and exogenous ligands.

SBDβ (substrate-binding domain) of GRP78, the domain responsible for interaction with substrates, was reported in previous study to bind with Pep42 (13 residues cyclic peptide, CTVALPGGYVRVC), GRP78 SBDβ residues (I426, T428, V429, V432, T434, F451, S452, V457, I459) recognize the cyclic peptide Pep42 and interact (bind) with it. This was used to deliver doxorubicin specifically to cancer cells that upregulate the cell-surface GRP78, enhancing targeted drug delivery while minimizing off-target toxicity^44–47^. The partial similarity and chemical compatibility of CRIPTO’s CFC domain regions with Pep42 are in accord with the proposal that these CRIPTO regions can bind GRP78 by conserved binding mechanisms that do not involve exact sequence homology.

The CFC domain (C115–C149)” CGSVPHDTWLPKKCSLCKCWHGQLRCFPQAFLPGC” of CRIPTO is a cysteine-rich domain, in order to obtain chains similar to Pep42 which might interact with GRP78 and then binding occurs, so we selected varied cystine-cystine “chins” regions from the CRIPTO’s CFC domain (4 regions) [region1 (C115–C128), region2 (C115–C133), region3 (C128–C140), region4 (C140–C149)]. The partial similarity and chemical compatibility of CRIPTO’s CFC domain regions with Pep42 are in accord with the proposal that these CRIPTO regions can bind GRP78 by conserved binding mechanisms that do not involve exact sequence homology. This enables us to study how GRP78 SBDβ residues specified residues will interact with every region from CFC domain of CRIPTO.

Multiple sequence alignment (MSA) was performed between each region of the CRIPTO CFC domain with the PEP42 peptide using the Clustal omega webserver (https://www.ebi.ac.uk/jdispatcher/msa/clustalo)^48^. ESpript 3 software was utilized to represent the results of the MSA (https://espript.ibcp.fr/ESPript/ESPript/)^49,50^. Then we used the ProtParam tool in the ExPASy (swiss bioinformatics resource portal) to compare the grand average of hydropathy (GRAVY) of the four regions of the CRIPTO CFC domain with the PEP42 peptide (https://web.expasy.org/protparam/)^51,52^.

GRP78 X-ray diffraction solved structure PDB file (PDB ID: 7N1R, Resolution: 2.03 Å) released in 2022 was downloaded from the protein data bank (PDB)^41,53^. Using PyMOL software we prepared the structure for the docking^54^. Water molecules, ions, ligands, and other chains of the protein were deleted keeping one chain in the PDB file. No solved structure for CRIPTO (cripto-1) protein was found in the protein data bank, so we used the ALPHAFOLD model (predicted structure) of CRIPTO (AF-P13385-F1-v4, https://alphafold.ebi.ac.uk/entry/P13385), the model was clean from any water or ions and has one chain^43,55^.

### Protein–protein docking

Protein–protein docking is one of the most relevant methods to study the interaction between proteins. In this study we use the HADDOCK web server (https://rascar.science.uu.nl/haddock2.4/), an integrative platform for the modeling of biomolecular complexes^56^. Also,^57^ there is ClusPro web server, a web server performs rigid-body docking of two proteins. we selected HADDOCK over ClusPro due to it’s improved handling of flexibility and inclusion of experimental/biophysical restraints. While ClusPro is extremely fast and efficient in the automated rigid-body docking, HADDOCK possesses a more sophisticated, data-driven approach with explicit flexibility (side-chain and backbone rotation) and optimization of solvation, and thus is superior at predicting complex biomolecular interactions. The energy-based scoring function and the ability to incorporate NMR, mutagenesis, or cryo-EM data of HADDOCK improve accuracy for our system. On the other hand, ClusPro’s excessive reliance on rigid-body docking and clustering, although computationally efficient in high-throughput screening, is not as highly granular as our research needs. HADDOCK was thus chosen based on its precision and flexibility against our experimental limitations^57–61^.

We performed protein–protein docking of GRP78 SBDβ selecting residues (I426, T428, V429, V432, T434, F451, S452, V457, I459) as active site with each region of the 4 regions determined in CRIPTO CFC domain using HADDOCK web server. The Prodigy web server was used to predict the binding affinity (Gibbs free energy) for each protein alone and the best cluster complexes with the highest haddock score of each region (https://rascar.science.uu.nl/prodigy/)^62^.

Then Protein–Ligand interaction profiler (PLIP) is used to detect the interactions between the proteins in all complexes (https://plip-tool.biotec.tu-dresden.de/plip-web/plip/index). PLIP provides a more detailed picture of all interactions at an atomic level, such as hydrogen bonding, hydrophobic interaction, and salt bridges which are extremely helpful in determining the residues responsible for the interaction between the two proteins^63^.

For visualization and representations of the protein complexes UCSF ChimeraX was used, a molecular visualization tool that provides high-quality 3D structures to clearly represent the critical interactions and interpret the PLIP data more effectively^64^.

### Molecular dynamics simulation

Molecular dynamics simulation (MDS) was performed for the four best score complexes of GRP78-CRIPTO using Groningen Machine for Chemical Simulations (GROMACS. 2021 version) a molecular dynamics simulation software utilizing CHARMM36 force field^65–67^.

The systems were prepared using the CHARMM-GUI (Chemistry at HARvard Macromolecular Mechanics) platform (https://www.charmm-gui.org/)^68^, solvating the system with the TIP3P water model and the ionization was by NaCl with the physiological concentration 0.154 M and PH = 7, at 310.15 K temperature.

The steepest descent technique was used to minimize the system, meaning that no atom encountered a force greater than 1000 kJ/mol/nm. In order to achieve a stable low-energy conformation free of high-energy areas and steric conflicts, this minimization was carried out for a maximum of 5000 steps. Equilibration was conducted in the NVT (constant number of particles, volume, and temperature) ensemble for 125 ps, and the temperature was maintained at 310.15 K using the V-rescale thermostat. Equilibration in molecular dynamics simulations ensures that the system is thermodynamically stable and consistent at the time of collecting data. Equilibration of the systems was confirmed after 125 ps run when these properties fluctuated around a mean value with minimal deviation. We confirmed the equilibration run after trajectory convergence was assessed as the physical properties like temperature (reached 310.15 K) and energy also volume of the system were stabilized.

Then a production simulation of 100 ns was carried out in the NPT ensemble. The Particle Mesh Ewald (PME) method is used for calculating long-range electrostatic interactions and the Coulomb interactions are truncated at 1.2 nm also, evaluating van der Waals interactions. A force-switch modifier is applied to smoothly turn off van der Waals interactions as atoms approach the cut-off distance (1.2 nm). The temperature was controlled the same as equilibration using the velocity-rescaling thermostat with a time constant of 0.1 ps. Pressure control was implemented with the C-rescale (stochastic cell rescaling) barostat with a time constant of 2 ps^69,70^, ensuring isotropic pressure coupling at 1 atm. During the production run, position restraints were removed from all atoms, allowing the system to relax fully. The analysis of the trajectories was obtained using the built-in modules of GROMACS.

### Gibbs free energy calculation

Free energy calculations are fundamental in structure-based drug design, as they provide quantitative predictions of the interactions between proteins and ligands. MMGBSA (Molecular Mechanics Generalized- Born Surface Area) and FEP (free energy perturbation) are good methods used in free energy calculations and has been employed in various studies^71–74^. Table 1. Clarify a comparison between FEP and MMGBSA methods. We employed MMGBSA over FEP due to it’s balance between speed and reasonable accuracy achieved through implicit solvation and ensemble-averaged MDS snapshots, as FEP is slow demands very high computational cost.

**Table 1:** Informative comparison between FEP and MMGBSA.

Feature	FEP	MM/GBSA	References
Accuracy	High (explicitly accounts for perturbations)	Moderate (relies on approximations)	^72,74–78^
Computational cost	Very high (requires multiple simulations)	Low (uses single or multiple MDS snapshots)	
Methodology	Perturbation-based (gradual transformation of states)	End-point method (energy decomposition of a single state)	
Solvation Model	Explicit solvent (more accurate)	Implicit solvent (GB/SA approximation)	
Dependence on MD	Requires extensive MD sampling for each perturbation	Can work with a single MD frame (but benefits from averaging)	
Speed	Slow (weeks to months for large systems)	Fast (hours to days)	

The binding free energy (Gibbs free energy) was calculated using the trajectory of the molecular dynamics simulation using MMGBSA calculations through the gmxMMPBSA tool. It computes contributions from van der Waals (VDW) interactions, electrostatic energies (EEL), solvation energies (EGB and ESURF), and entropy corrections. Energy decompositions, including bond, angle, and dihedral terms, are also provided. Conformational snapshots were extracted from the trajectory over a specified time window, and energy contributions were computed for each complex. The electrostatic solvation was calculated using the Poissson-Boltzmann equation, while the non-polar solvation energy was derived from the solvent-accessible surface area.

## Results and discussion

The cyclic peptide Pep42 was used to target the SBDβ of GRP78 overexpressed on cancer cell membranes in previous studies^79,80^. This peptide was utilized in the prediction of GRP78 SBDβ targeting in many viral and fungal infections^47,81–86^.

**Fig. 1:**
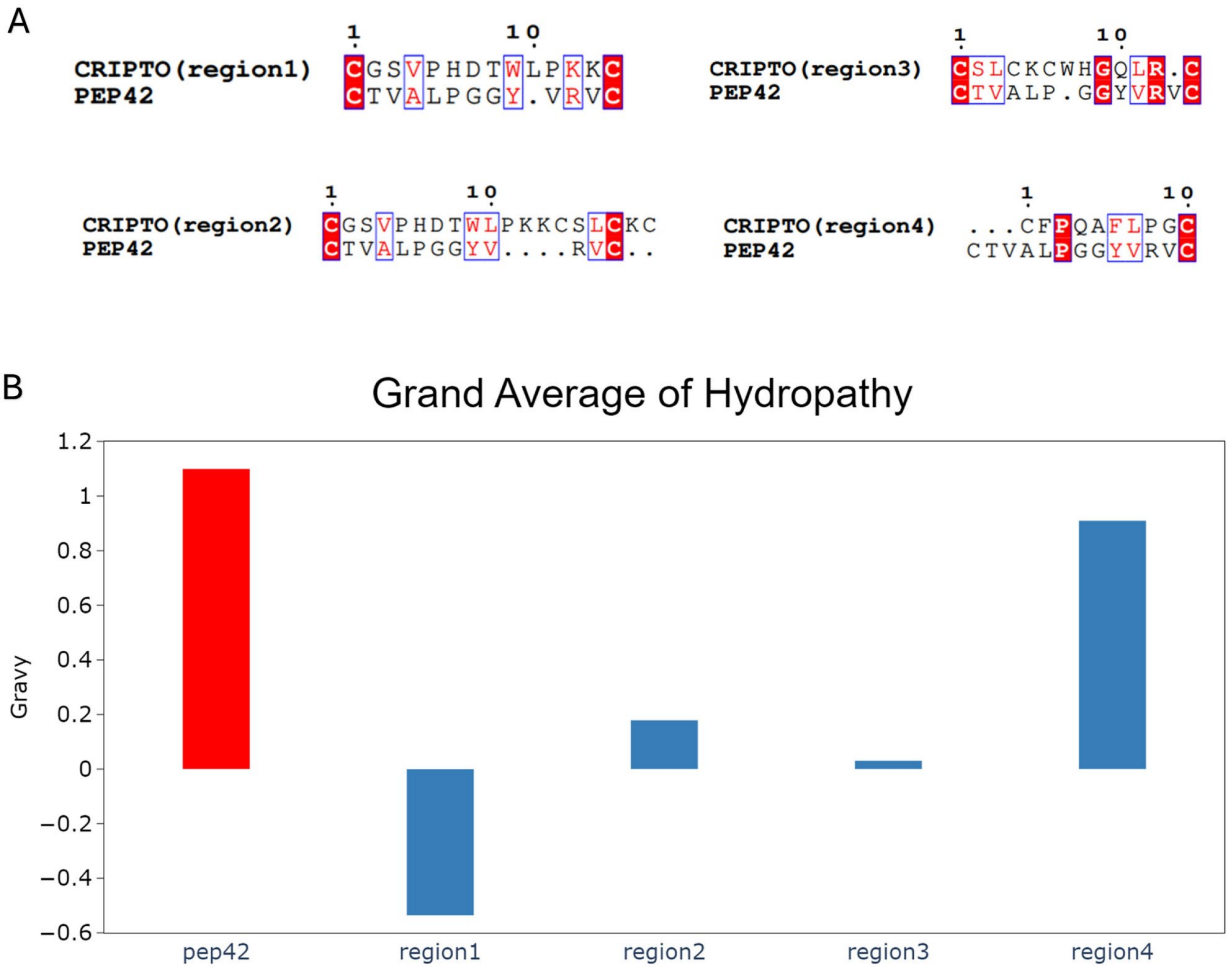
(**A**) The pairwise sequence alignment between the cyclic peptide pep42 and the four different regions of the CRIPTO CFC domain. (**B**) The grand average hydrophobicity index (GRAVY) of the four different regions of the CRIPTO CFC domain (blue) compared to the GRAVY of the Pep42 peptide (red).

Multiple sequence alignment of all regions of the CRIPTO CFC domain with Pep42 is clarified in Fig. 1A. The obtained alignment indicated 15% sequence identity between the CRIPTO CFC domain region1 and Pep42, 30% in region2, 15% in region3, and 20% in region4. Although these values are not high, it does not rule out functional relevance. Especially in protein–protein interactions where short segments of peptides or domains are involved, high sequence similarity has long been known not to be a prerequisite for binding to occur, and the identity values obtained in the sequence comparison suggest that these regions could still interact favorably with GRP78 SBDβ. This is because binding is more dependent on conserved physicochemical features, including charge distribution, hydrophobicity, and secondary structure potential, than on residue-by-residue match. Pep42 is a cyclic peptide that binds to GRP78 via hydrophobic interaction and shape complementarity.

The partial sequence homology seen between CRIPTO’s CFC domains like hydrophobic and aromatic residues (Leu, Phe, Val) may perhaps be able to approximate the important interaction motifs present in Pep42. Moreover, proteins with 25% sequence identity have been reported to possess structural and functional homologies, particularly when short functional motifs or domains are compared^87^. Therefore, even at low sequence identity, sequence similarity between those regions of the CRIPTO CFC domain and Pep42 suggests structural and functional homology, and the CRIPTO CFC fragments are legitimate candidates for GRP78 binding based on the overall chemical nature and motif-like quality of the regions under consideration.

The presented data therefore raises their potential relevance for GRP78-mediated processes and provides further study regarding the affinity and interaction mode with the candidate fragments (regions) of the CRIPTO CFC domain.

Hydropathy profiles (see Fig. 1B) of Pep42 against the four regions of the CRIPTO CFC domain (region1, region2, region3, and region4) show distinct differences in hydrophobicity as determined by their GRAVY scores. Pep42 is highly hydrophobic, with a GRAVY score of 1.1, indicating strong hydrophobic interactions. On the other hand, the region1 of the CRIPTO CFC domain is hydrophilic, with a GRAVY score of − 0.536, reflecting weaker hydrophobic binding. Whereas region 2 of the CRIPTO CFC domain has a GRAVY score of − 0.179, slightly less hydrophilic but still predominantly hydrophilic. Similarly, region 3 displays a GRAVY of approximately 0.031, reflecting near neutrality but with moderate hydrophobicity, and would possibly interact with hydrophobic or hydrophilic regions. Contrary, region 4 of the CRIPTO CFC domain bears a GRAVY score of approximately 0.910, high hydrophobicity, just as Pep42, and probably binds strongly at hydrophobic sites. Based on this parameter, it looks very much alike in hydrophobic profile and binds similarly to its counterpart, and similarly placed regions 1, 2, and 3 are somewhat hydrophilic and should interact differently.

### Protein–protein docking

#### Multiple sequence alignment and GRAVY calculation

The protein–protein docking results show that the 4 selected regions from the CRIPTO CFC domain make strong interactions with GRP78 SBDβ with a HADDOCK score of − 70.4, − 78.7, − 74.2, and − 86.8 & prodigy predicted binding affinity − 8.5 kcal/mol, − 8.3 kcal/mol, − 8.5 kcal/mol, and − 7.9 kcal/mol for complex1 (region1), complex2 (region2), complex3 (region3), complex4 (region4) respectively. Relating this docking scores with hydropathy profiles by GRAVY scores provide additional insight into the likelihood of interaction between GRP78 and CRIPTO. Pep42 has a large positive GRAVY score (1.1) and has been shown to strongly interact with GRP78’s SBDβ hydrophobic patches. Region 4 of the CRIPTO CFC domain is also highly positively scored by GRAVY (0.910), and this was matched with the highest of the four complex HADDOCK scores (− 86.8). On the other hand, regions 1 and 2, the more hydrophilic (GRAVY scores − 0.536 and − 0.179, respectively), had lower docking scores. This is consistent with hydrophobicity’s primary role in stabilizing the interaction by allowing van der Waals contacts and reduced solvation energy in the binding interface. These findings establish the utility of hydropathy profiling as a predictive assay in evaluating protein–protein binding propensity. This indicates that all the regions selected for the CRIPTO CFC domain will show good interactions with the GRP78 SBDβ. Table 2 shows The PLIP analysis report for the four CFC regions and the interactions occurring between the CRIPTO CFC domain and GRP78 SBDβ such as Hydrogen bonds, hydrophobic interactions, and salt bridges.

**Table 2:** HADDOCK score and Prodigy binding affinity ΔG in (kcal mol^−1^). The PLIP analysis data of the best HADDOCK clusters for the 4 regions of CRIPTO docked with GRP78 shows the formed Hydrogen bonds, hydrophobic interactions, and salt bridges between the two proteins (GRP78-CRIPTO) for each complex.

PLIP interactions, HADDOCK, and Prodigy data											
Complex	Haddock score	Prodigy binding affinity ΔG (kcal mol^−1^)	H-bonds			hydrophobic			Salt bridges		
			No	CRIPTO amino acids	GRP78 amino acids	No	CRIPTO amino acids	GRP78 amino acids	No	CRIPTO amino acids	GRP78 amino acids
Complex1 (region1)	− 70.4	− 8.5	7	P117, W123(2), P125(2), K126, and Q143	G430, G431(2), Q449, S452(2), and Q492	7	P39, T122, W123(2), L124, P125, and L130	L417, V432(3), I450(2), and F451	3	R38(3)	E217, D238, and E243
Complex2 (region2)	− 78.7	− 8.3	11	K126, C128, S129(2), Q137, C140, G151, R162, and T163	S452(2), V453, T456(4), 479, 481, and 488(2)	1	L138	V453	2	D525 and D479	K127(2)
Complex3 (region3)	− 74.2	− 8.5	10	I33, Q34, E35(2), R38(2), W123, P125(2), and Q143	T236, E243, D350(4), G431, T434, and S452(2)	4	W123(2), P125, and L130	V432(3) and F451	1	R38	E217
Complex4 (region4)	− 86.8	− 7.9	7	D2, K5, W123, Q143, A144(2), and D150	T434, Q449, S452, R488, N539, and E542	4	W123, L130, and F145(2)	F451(2) and V490(2)	2	K5 and D121	R488 and E542

**Fig. 2:**
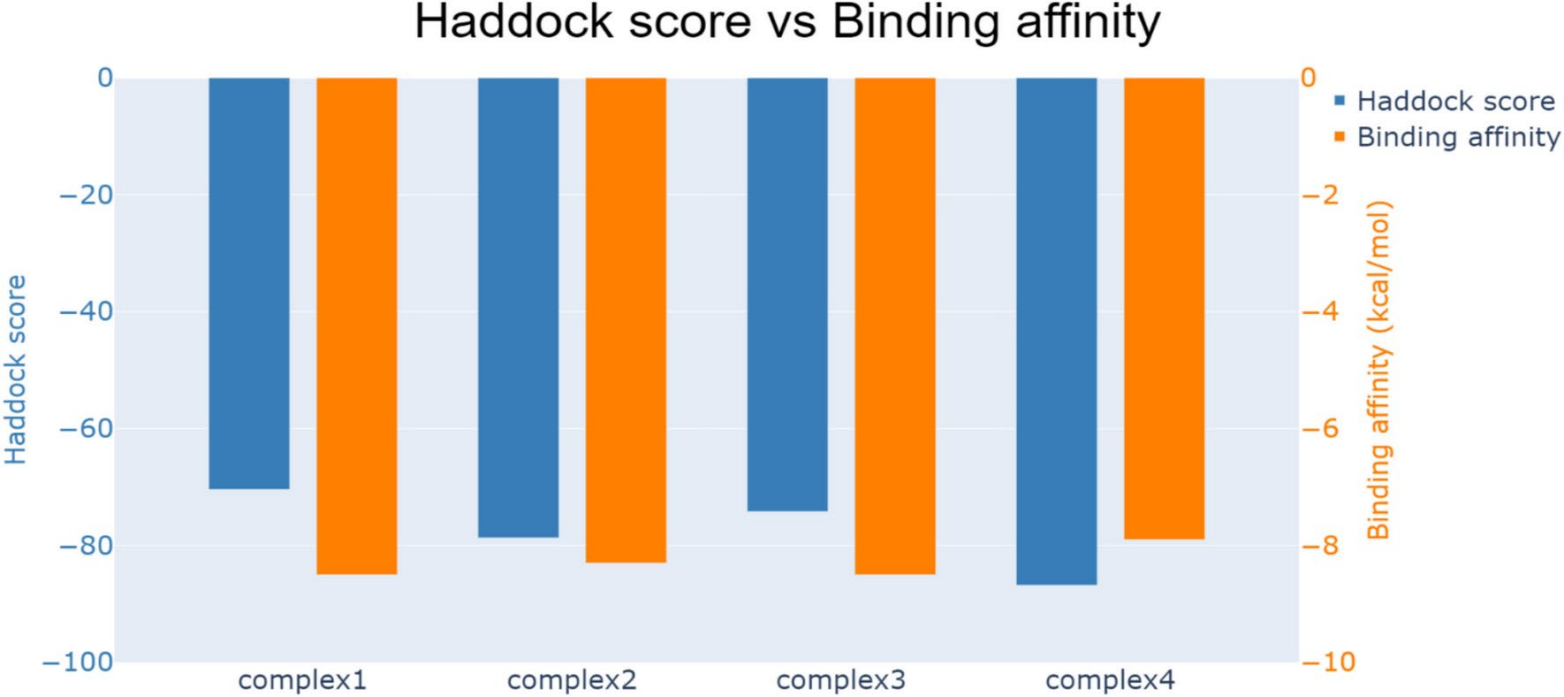
HADDOCK score (blue bar) and prodigy binding affinity (in kcal/mol) (orange bar) of the 4 complexes (GRP78-CRIPTO).

**Fig. 3:**
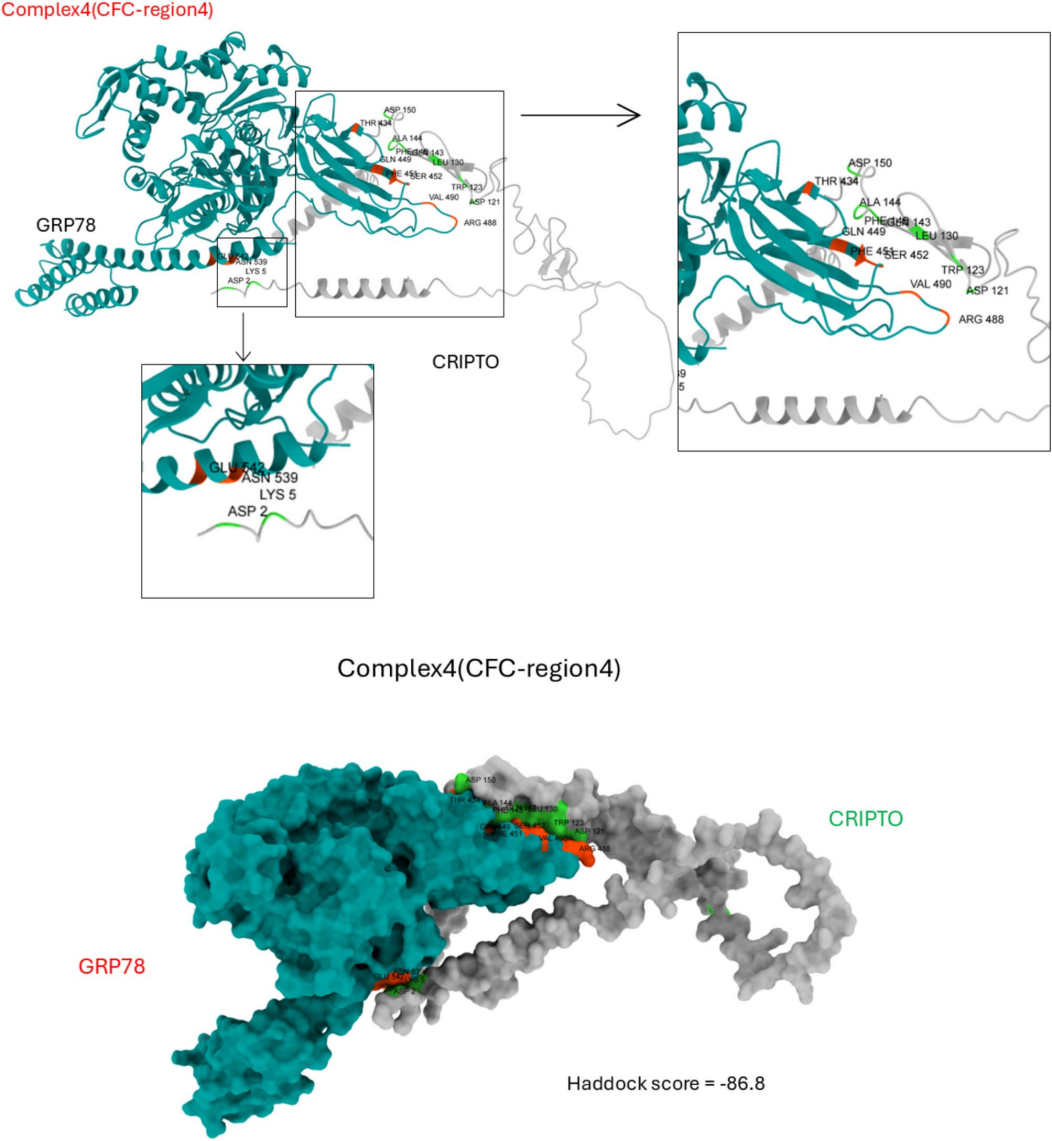
Complex4 (best haddock score) representations, GRP78 interacting residues(orange) and CRIPTO interacting residues(green).

Figure 2 shows the HADDOCK scores and prodigy binding affinity (Gibbs free energy) of the best modes of best clusters of each complex (due to 4 CRIPTO CFC regions). For complex1, there are; 7 hydrogen bonds between GRP78 (G430, G431(2), Q449, S452(2), and Q492) and CRIPTO (P117, W123(2), P125(2), K126, and Q143), 7 hydrophobic interactions between GRP78 (L417, V432(3), I450(2), and F451) and CRIPTO (P39, T122, W123(2), L124, P125, and L130), and 3 salt bridges between GRP78 (R38(3)) and CRIPTO (E217, D238, and E243). On the other hand, the complex2 had 11 hydrogen bonds, one hydrophobic interaction, and two salt bridges between the two proteins. Also found in complex3 10 hydrogen bonds, 4 hydrophobic interactions, and one salt bridge. For complex 4 (the best HADDOCK score complex, shown in Fig. 3) 7 hydrogen bonds, 4 hydrophobic interactions, and two salt bridges are formed. All the amino acids involved in all the interactions formed between the two proteins in every complex are listed in Table 2.

The specificity and stability of the GRP78-CRIPTO complex are primarily governed by pivotal interactions like hydrogen bonds, salt bridges, and hydrophobic interaction which exhibited in all the four regions interaction of CRIPTO’S CFC domain with GRP78 SBDβ. Hydrogen bonds are responsible for orienting the proteins and stabilizing the binding interface, and electrostatic stability in the nature of salt bridges stabilizes the complex, contributing to additional strength to the interaction. Hydrophobic interactions contribute their share by minimizing solvent exposure of the nonpolar groups, and thus maximizing the production of a more stable, energetically favorable complex. Collectively, these interactions synergize to generate a robust binding affinity, highlighting their importance to the complex’s overall stability and specificity. These molecular forces are required to clarify the mechanism of binding and provide valuable insights of the GRP78-CRIPTO interaction.

### Molecular dynamics simulation

**Fig. 4:**
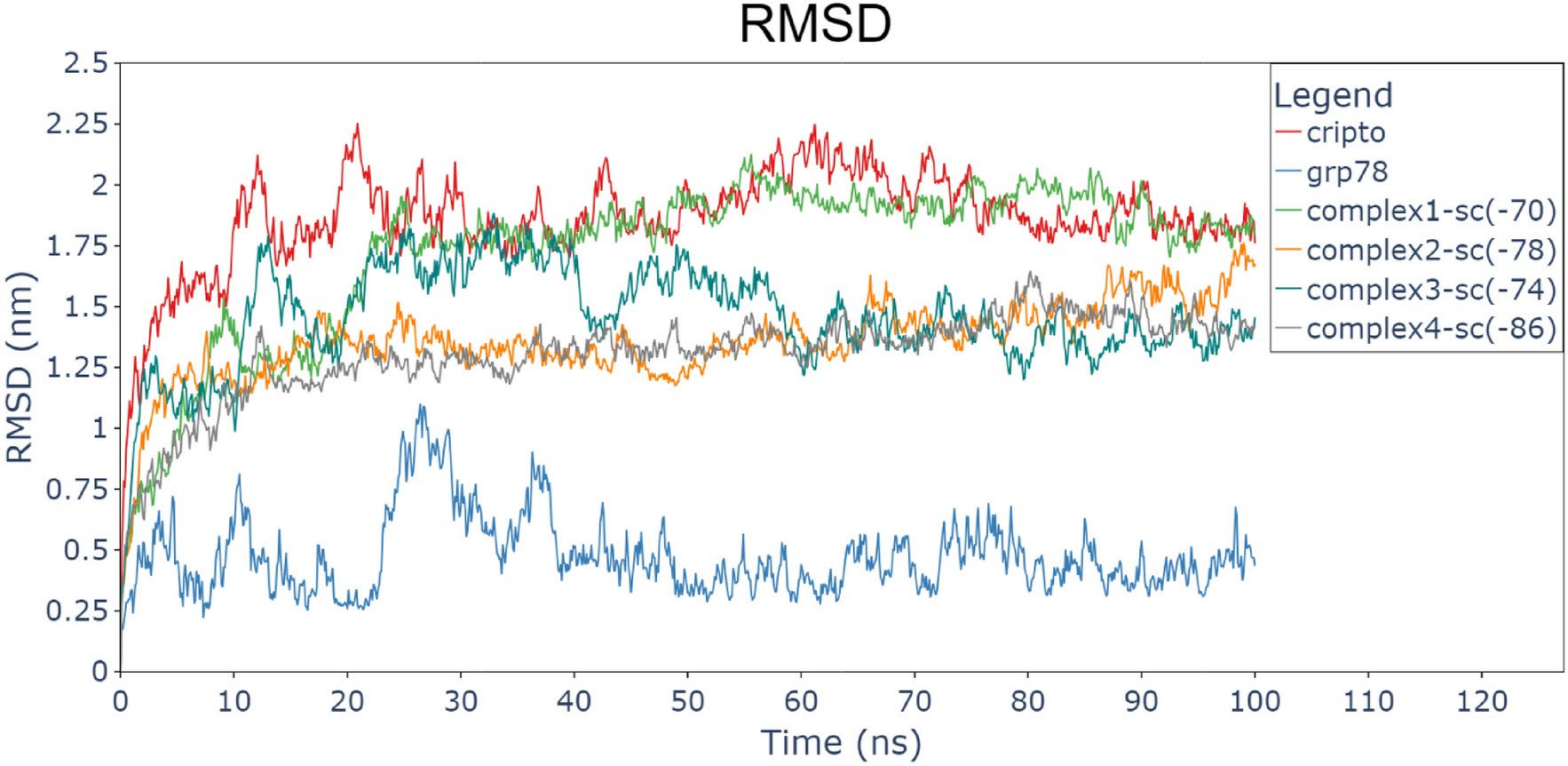
Root-mean-square deviation (RMSD) for each protein and the 4 complexes (GRP78-CRIPTO). CRIPTO (red), GRP78 (blue), complex1 (green), complex2 (orange), complex3 (sea green), complex4 (gray).

The systems equilibrated during the 100 ns runs; the GRP78, CRITPO, and the complexes (1, 2, 3, and 4). Figure 4 shows time evolution in nanoseconds for root mean square deviation (RMSD) for each protein simulated alone and the four complexes. This indicates that GRP78 (blue) had lower RMSD compared to CRIPTO (red). This pattern is due to the secondary structure of the proteins. GRP78 contains a lot of alpha helices and beta sheets and less number of loops (a short, flexible region in a protein that connects more rigid structures like α-helices and β-sheets), unlike CRIPTO which is a small protein that contains a more loops that make the protein more flexible and enabled CRIPTO to has interactions within its domains and terminuses. Although the nature of loops makes them high fluctuating during simulations, this dynamic character can influence the stability of the docking process and stabilize the overall complex by facilitating more conformations of the protein and during the docking, also key interactions such as hydrogen bonds, salt bridges, and hydrophobic contacts. Despite the flexibility, long-term stability of the GRP78-CRIPTO complex is provided by a combined action of these interactions. So the effect of loop flexibility is critical to enhance docking models and make predictions on binding affinity and specificity in protein–protein interactions.

The two terminuses of CRIPTO interacted (by attraction) with each other during the first 30 ns, then it became stable (equilibrated) during the next 70 ns taking an attracted pose between each other. RMSD for complex4(gray) is lower slightly than that of Complex2(orange) which has lower RMSD than complex1(green) and Complex3(sea green). This means that all the four regions of the CFC domain in CRIPTO makes interaction, and region 4 makes the best contact (binding) with GRP78 SBDβ (substrate-binding domain β).

**Fig. 5:**
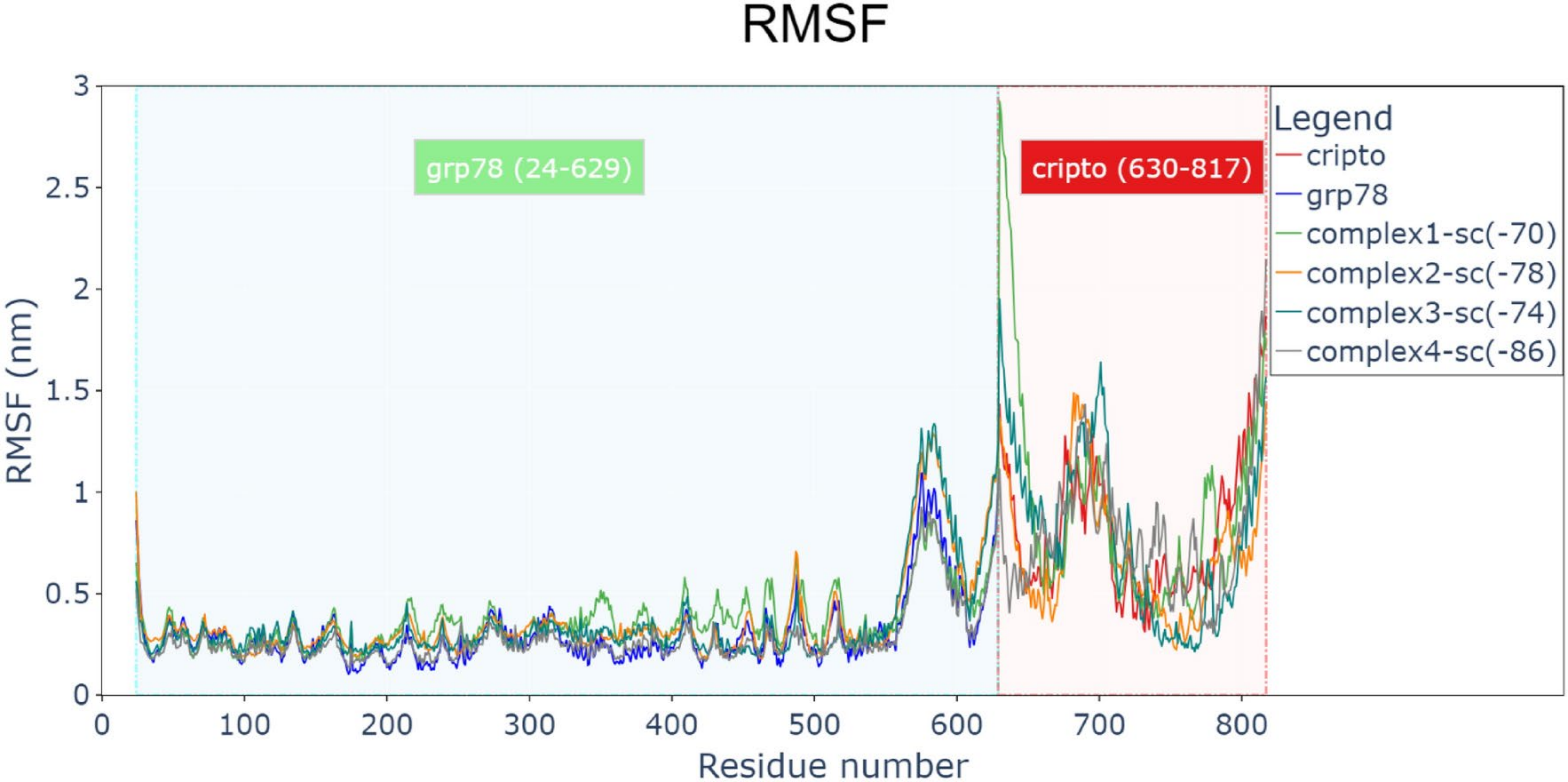
Root-mean-square fluctuation (RMSF) for residues of each protein and the 4 complexes (GRP78-CRIPTO). CRIPTO (red), GRP78 (blue), complex1(green), complex2(orange), complex3(sea green), complex4(gray).

**Fig. 6:**
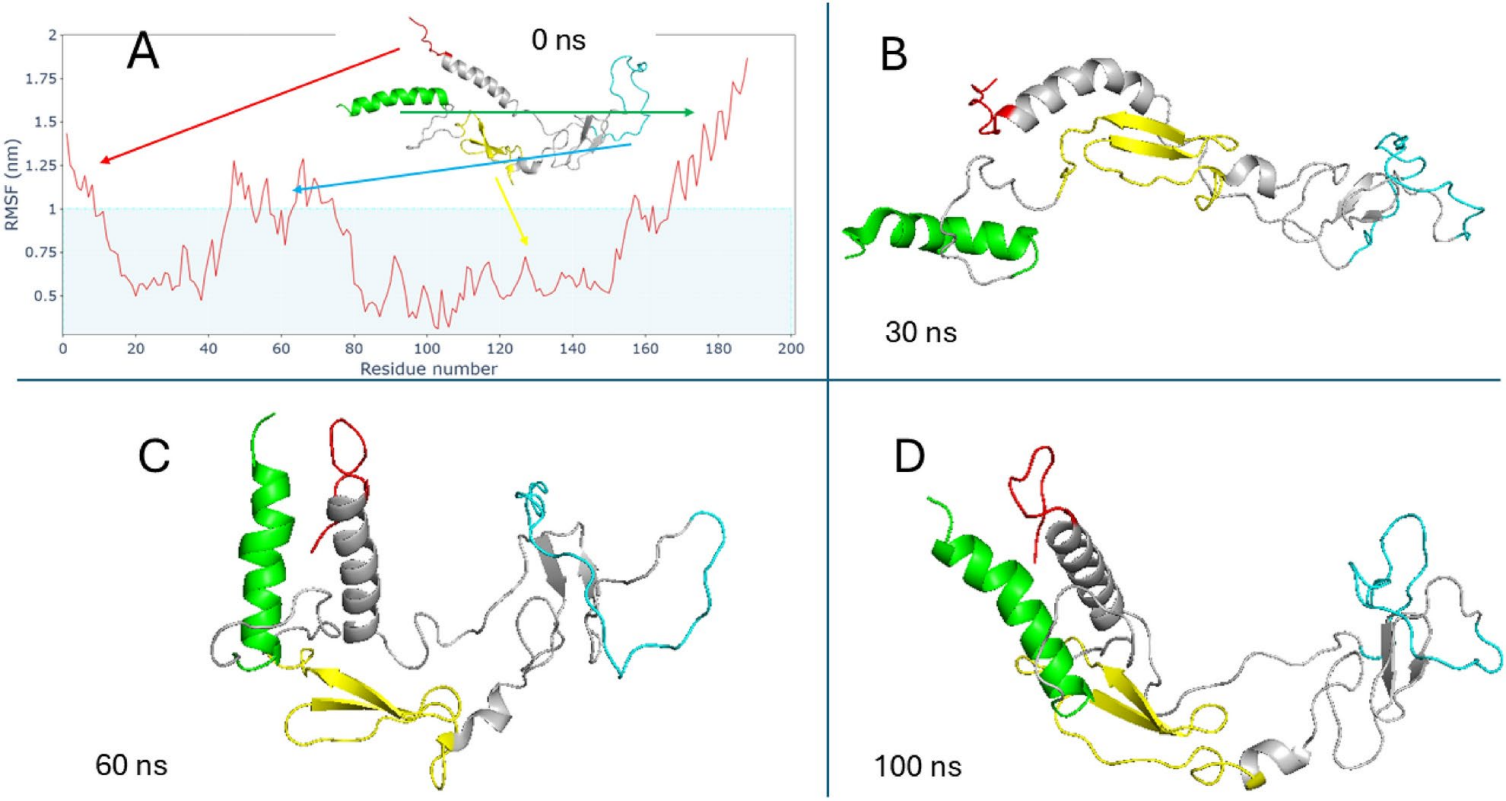
(**A**) residual RMSF of CRIPTO and CRIPTO conformation at 0 ns of MDS, N-terminus(red), C-terminus(green), CFC domain (yellow) and flexible part of loop(cyan). (**B**) CRIPTO conformation at 30 ns of MDS. (**C**) CRIPTO conformation at 60 ns of MDS. D) CRIPTO conformation at 100 ns of MDS.

Figure 5 shows root-mean-square fluctuation (RMSF) in nanometers for all residues of each protein and the complexes. Figure 6 clarifies the two terminuses of CRIPTO before and during the interaction (by attraction) with each other, N-terminus “red part, residues [M1–S10]” will interact during the MDS as clarified also in (Fig. 4) with C-terminus “green part, residues [L166–Y186]” making high RMSF values (more than 1 nm). Also, a loop part of CRIPTO “cyan part, residues [F46–S75] exhibited fluctuation (more than 1 nm). This enables us to see which part (residues) of any protein is responsible for the fluctuations or conformational changes. CRIPTO alone or in all complexes shows higher fluctuations than GRP78, especially the terminals of the protein and the loops in it as clarified, but the complexes still exhibit binding during the MDS time.

**Fig. 7:**
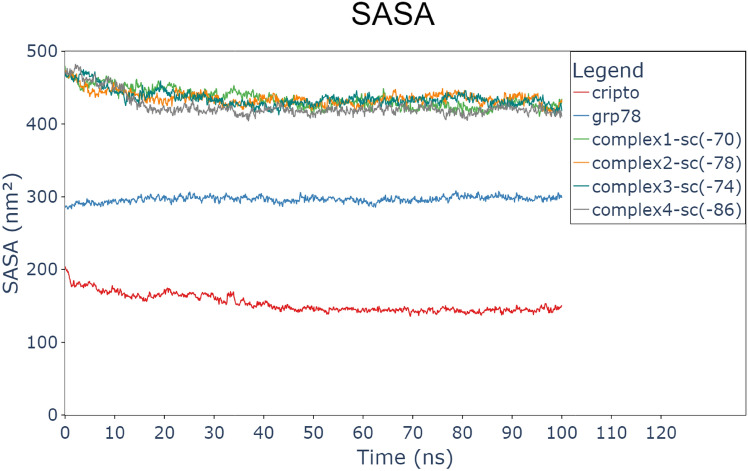
Surface accessible surface area (SASA) for every protein and the 4 complexes (GRP78-CRIPTO). CRIPTO (red), GRP78 (blue), complex1(green), complex2(orange), complex3(sea green), complex4(gray).

**Fig. 8:**
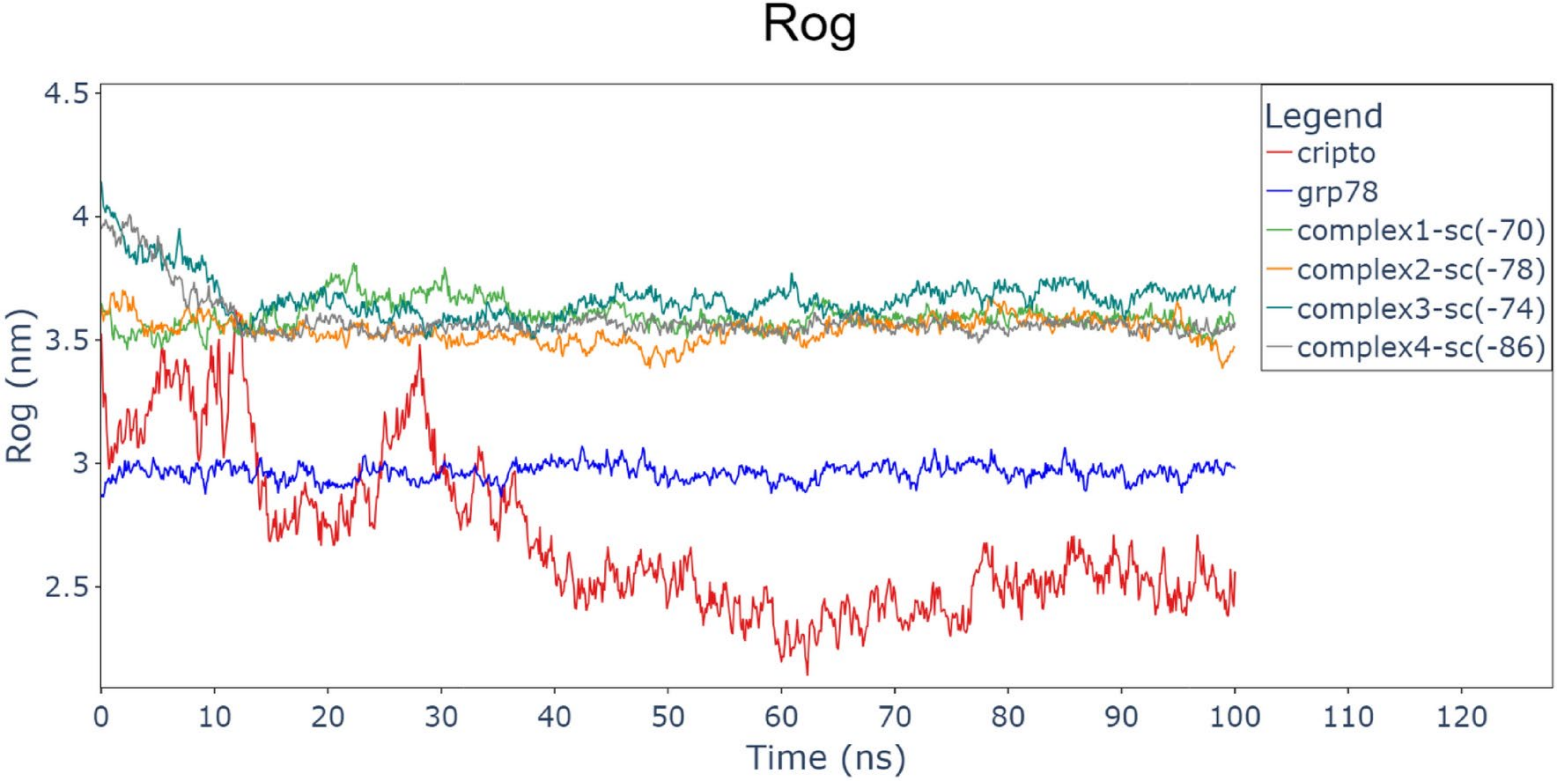
Radius of gyration (Rog) every protein and the 4 complexes (GRP78-CRIPTO). CRIPTO (red), GRP78 (blue), complex1(green), complex2(orange), complex3(sea green), complex4(gray).

Figures 7 and 8 show the surface accessible surface area (SASA) in nm^2^ and the radius of gyration (RoG) in nm, respectively. The SASA values indicate stable protein systems (GRP78 “around 300 nm^2^”, CRIPTO “around 150 nm^2^ “, and the four complexes “closely between 410 to 450 nm^2^) after a 20 ns equilibration period. The decrease in SASA indicates the reduction in ligand accessibility but also reflects the creation of a tighter and more stable binding interface between GRP78 and CRIPTO. Complex 4 was the lowest SASA and most stable and compact structure.

**Fig. 9:**
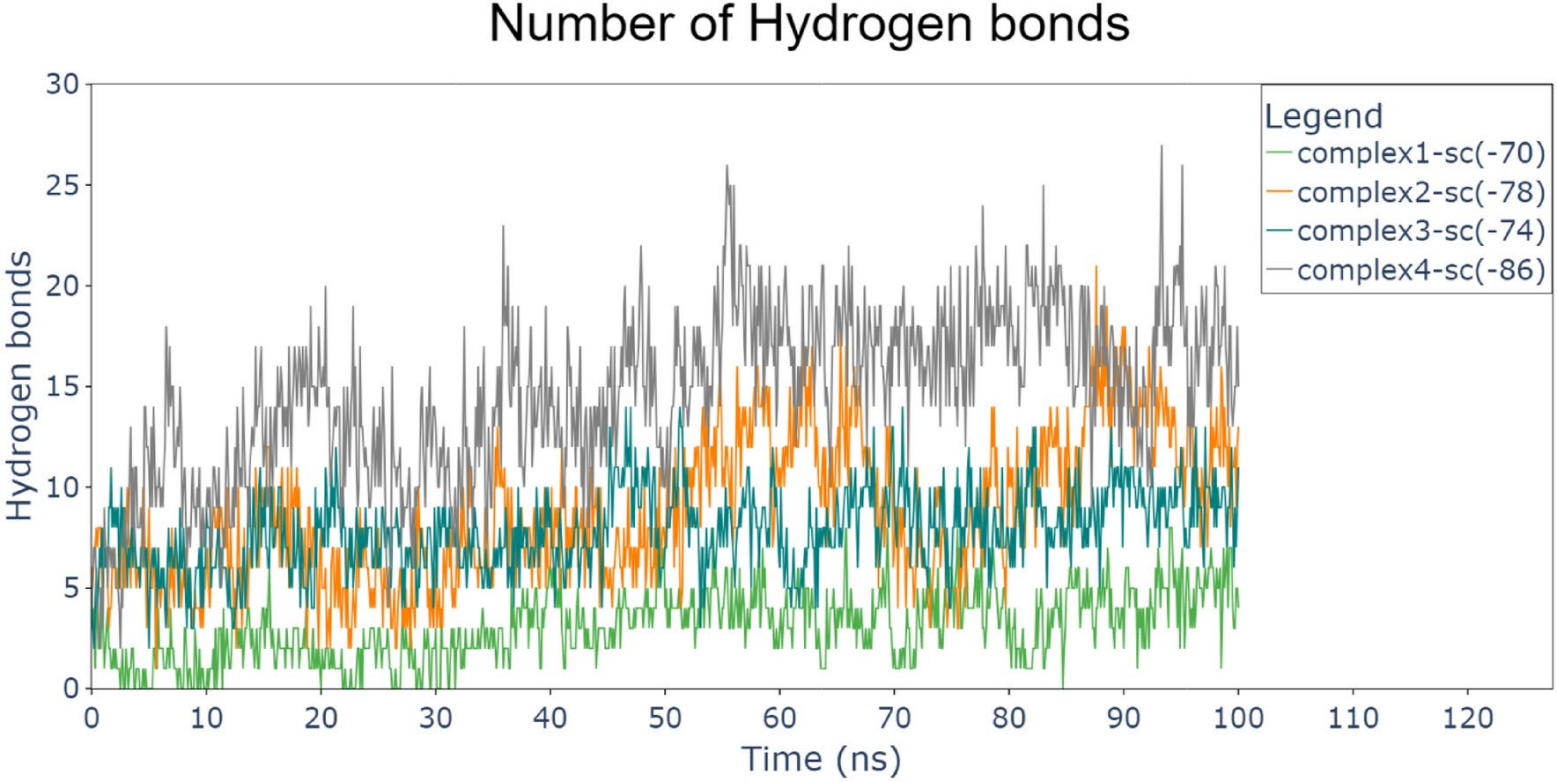
Hydrogen bonds between the two proteins in the 4 complexes (GRP78-CRIPTO). CRIPTO (red), GRP78 (blue), complex1(green), complex2(orange), complex3(sea green), complex4(gray).

Also, the radius of gyration stayed stable for GRP78 around 3 nm, implying that GRP78’s structure was relatively unaffected by the binding interaction. Also, CRIPTO RoG values decreased from 3.5 to 2.5 and became stable after 40 ns, this is due to the interaction between the two terminals of CRIPTO during this period. On the other hand, RoG of the four complexes (GRP78-CRIPTO) was stable between closely 3.5 nm and 3.8 nm. This reveals that all complexes equilibrated, and the systems reached their thermodynamic equilibrium during the simulation period. The RoG trends confirm that binding is linked with conformation tightening, particularly for GRP78, that becomes more compact to a stable conformation as well as the four complexes decreased to or stayed at a stable value. These observations supports the idea that binding between GRP78 and CRIPTO results in a conformational tightening and the most stable complex (Complex 4) being the lowest SASA and most tightly bound structure showing the most optimal binding mode (Fig. 9).

### Gibbs free energy calculation

The results of MM-GBSA calculations reveal the free binding energies of the four complexes as shown in Table 3. Complex 4 presents the strongest binding with a value of − 126.26 kcal/mol, followed by complex 3 (− 81.92 kcal/mol), complex 2 (− 59.78 kcal/mol), and finally complex 1 (− 15.07 kcal/mol). So, complex 4 has the most favorable free binding energy and stability due to the strongest Van der Waals (− 199.87 kcal/mol) and electrostatic interactions (− 1293 kcal/mol), which are highly contributory to its overall binding energy.

**Table 3:** The MM-GBSA calculations for the four complexes (GRP78-CRIPTO) after 100 ns molecular dynamics simulation. Van der Waals energy, electrostatic energy, generalized born, solvent accessible surface area energy, solvation free energy, and total free energy change components are listed.

Energy Component	Complex1 (CFC-region1/GRP78)	Complex2 (CFC-region2/GRP78)	Complex3 (CFC-region3/GRP78)	Complex4 (CFC-region4/GRP78)
ΔE VDWAALS (kcal/mol)	− 55.13	− 104.69	− 114.70	− 199.87
ΔE ELE (kcal/mol)	− 637.10	− 984.09	− 723.78	− 1293.61
ΔE GB (kcal/mol)	684.65	1043.95	773.50	1396.95
ΔE SURF (kcal/mol)	− 7.50	− 14.94	− 16.94	− 29.73
ΔG GAS (kcal/mol)	− 692.22	− 1088.79	− 838.49	− 1493.48
ΔG SOLV (kcal/mol)	677.15	1029.01	756.56	1367.21
ΔTOTAL (kcal/mol)	− 15.07	− 59.78	− 81.92	− 126.26

Table 4. shows the contribution of every individual residues (below − 1 energy values) of GRP78 and CRIPTO (Complex4) in the binding energy, clarifying the significant role of these residues in the over all stability of the interaction. residues such ARG:488 in GRP78 and ARG:8 in CRIPTO exhibited best binding energy contributions (− 6.006 kcal/mol and − 6.685 kcal/mol, respectively), also values in VAL:432 in GRP78 (− 3.218 kcal/mol) and PHE:145 (− 4.387 kcal/mol) in CRIPTO showed high contribution. The SBD residues of GRP78 (VAL:432, THR:434) and CFC’s residues of CRIPTO (PHE:145, TRP:123, PRO:147, THR:122) Also were crucial in the interaction which indicates to the importance of these residues (binding sites) in the stability of binding. Moreover, While Residues ARG 4, LYS 5, ARG 8, PHE 9, SER 10, LEU 31, LUE 152, LEU 158 and ARG 162 which lies in the most flexible part of the protein “loops”, it’s contribution to the binding of CRIPTO with GRP78 was superior and effective. This also confirms what’s discussed above, that specific loop residues have a pivotal role in the mediating protein–protein interactions.

**Table 4:** MMGBSA calculated residual contributions to binding for the Complex4.

complex	Complex4			
	GRP78’S residue	Binding energy (kcal/mol)	CRIPTO’S residues	Binding energy (kcal/mol)
Residual contribution to binding	ARG:488	− 6.006	ARG:8	− 6.685
	VAL:432	− 3.218	PHE:145	− 4.387
	PRO:487	− 2.980	ARG:162	− 3.959
	ILE:520	− 2.747	LEU:31	− 3.881
	PRO:531	− 2.581	LEU:152	− 3.663
	GLU:542	− 2.430	ARG:4	− 3.613
	PRO:485	− 2.370	MET:17	− 3.043
	THR:514	− 2.048	TRP:123	− 2.658
	VAL:453	− 1.816	VAL:13	− 2.625
	LYS:447	− 1.764	LEU:27	− 2.516
	PRO:484	− 1.741	PHE:9	− 2.348
	ASN:516	− 1.613	LYS:21	− 2.211
	ASN:539	− 1.566	LYS:5	− 1.897
	ALA:486	− 1.533	PHE:23	− 1.743
	ILE:483	− 1.527	PHE:175	− 1.623
	GLY:515	− 1.460	ILE:16	− 1.611
	VAL:538	− 1.449	SER:10	− 1.587
	LEU:417	− 1.361	PRO:147	− 1.452
	GLY:431	− 1.285	LEU:158	− 1.047
	THR:189	− 1.250	THR:122	− 1.0006
	THR:434	− 1.180		
	ILE:534	− 1.076		
	VAL:490	− 1.054		

Also the calculations demonstrates that complex 4 exhibits the highest binding affinity and thermodynamic stability and the best Van der Waals and electrostatic interactions contributing significantly to its binding energy. Despite a higher polar solvation penalty, the favorable non-polar solvation effects, particularly due to hydrophobic interactions, are enough to offset this cost and contribute to the most negative total free energy. The comparative stabilities of the complexes 1, 2, and 3 are a result of progressively decreasing binding energies due to progressively weaker non-polar solvation contributions and reduced interaction energies. The balance between polar and non-polar solvation contributions has an important role in assessing the stability of the complexs, Complex 4 depends on the presence of the stronger hydrophobic interaction along with stronger interaction forces compared to all others, as it’s attractive forces within complex 4 outweigh the desolvation penalties, leading to the most negative total free energy. This would indicate that all four regions of the CRIPTO CFC domain showed good interaction with GRP78 SBDβ. Complex 4 is the most thermodynamically stable and tightly bound complex but complex 1 indicates the weakest binding affinity.

The aforementioned reasons make complex 4 the most promising candidate to be studied further, especially concerning drug design or molecular interaction studies in future work.

#### Previous studies compared with our novel findings.

An in-silico study performed on the structures of GRP78 and CFC domain of (CFC only, sequence form mouse) utilizing molecular docking and MDS, revealed that CRIPTO CFC domain interacted and stabilized GRP78 into the cell membrane as CRIPTO’S CFC bind GRP78 NBD^88^.

Experimental studies also conducted in the last 3 decades ensures the importance of targeting GRP78 and CRIPTO role in cancers as they play crucial role in cancer proliferation, maintenance and therapy resistance^30^. The interaction between GRP78 NBD and CRIPTO CFC was studied and confirmed in NCCIT “extracted from human embryonal carcinoma cell lines” and MCF 10A cell lines, proving CRIPTO binding to cell surface GRP78 (CSGRP78) is crucial for the signaling of CRIPTO in human tumors, revealing that CRIPTO/GRP78 complex critically regulates TGF-β signaling, promoting tumor proliferation and suppressing the tumor fighting effect of activin-A and TGF-β1. shRNA knockdown and GRP78-blocking N-20 antibodies also anti-actin antibodies disrupted the interaction and inhibited PI3K/Akt, MAPK/ERK, and c-Src activation^28,30,89^.

Monoclonal antibodies were used to inhibit CRIPTO’s CFC and EGF domains (EGF (A27.F6.1 antibody) and CFC (A8.G3.5 antibody)) in NCCIT testicular and colon carcinoma cell lines xenografted in mice. Results showed that (A8.G3.5) antibody achieved up to 70% tumor growth suppression^90^.

Also GRP78 was targeted in NSCLC (non-small cell lung cancer) and GBM (glioblastoma multiforme) with polyclonal and monoclonal antibodies, eliciting selective apoptosis of cancer cells after prolonged treatment (72–96 h), inhibiting central Akt/mTOR survival signaling pathways, and greatly sensitizing for radiation response in vivo and in vitro. Notably, these actions were cancer cell-specific and did not impact normal cells^91^.

All these findings confirm the previous computational and experimental studies performed to detect the interaction between GRP78 and CRIPTO proteins proved that the complex drives cancer progression by promoting tumor cell survival, growth, and metastasis. Its overexpression in tumors makes it a promising therapeutic target for anticancer strategies.

Our work reveals novel results confirming these studies, as we suggesting new binding sites between GRP78 SBDβ (I426, T428, V429, V432, T434, F451, S452, V457, I459) residues and CRIPTO’s CFC domain (all regions selected, region4 is best). Paving the way for targeting these domains with new drugs or antibodies to inhibit cancer progression, resistance and maintenance.

## Conclusion

Glioblastoma is one of the most malignant cancers for the central nervous system (CNS), accounting for 14.5% of CNS tumors with an overall survival median of around 15 months. This is very concerning, as it was reported that GRP78 and CRIPTO overexpression in the cancer cells activate MAPK/AKT signaling, Src/PI3K/AKT, and Smad2/3 pathways leading to tumor proliferation, plasticity, and resistance to apoptosis. To deactivate these pathways, so we have to inhibit the complex formation of the two proteins. Our study, using molecular docking and molecular dynamics simulation methods, elucidates that the interaction between CRIPTO and GRP78 is possible in new suggested binding sites, as SBDβ of GRP78 recognizes and binds tightly with the CFC domain of CRIPTO. We predicted the binding site in the CRIPTO CFC domain responsible for the interaction with specific residues of GRP78 SBDβ which can be targeted both by inhibitors in future work to prevent this interaction leading to stopping the pathway responsible for cancer resistance to chemotherapy.

## Data Availability

Data generated during this study is included in the manuscript.
